# Clinical effects of curcumin in enhancing cancer therapy: A systematic review

**DOI:** 10.1186/s12885-020-07256-8

**Published:** 2020-08-24

**Authors:** Kamran Mansouri, Shna Rasoulpoor, Alireza Daneshkhah, Soroush Abolfathi, Nader Salari, Masoud Mohammadi, Shabnam Rasoulpoor, Shervin Shabani

**Affiliations:** 1 Medical Biology Research Centre, Kermanshah University of Medical Sciences, Kermanshah, Iran; 2 Department of Biology, Islamic Azad University Urmia, Urmia, Iran; 3 School of Computing, Electronics and Maths, Coventry University, Coventry, UK; 4 Centre for Predictive Modelling, University of Warwick, Coventry, CV4 7AL UK; 5 Department of Biostatistics, School of Health, Kermanshah University of Medical Sciences, Kermanshah, Iran; 6 Sleep Disorders Research Center, Kermanshah University of Medical Sciences, Kermanshah, Iran; 7 Department of Nursing, School of Nursing and Midwifery, Kermanshah University of Medical Sciences, Kermanshah, Iran; 8 Department of Nursing, School of Nursing and Midwifery, Kermanshah University of Medical Sciences, Kermanshah, Iran; 9 Department of Biology, Islamic Azad University Urmia, Urmia, Iran

**Keywords:** Prevalence, Curcumin, Clinical feature, Cancer, Systematic review

## Abstract

**Background:**

Curcumin is herbal compound that has been shown to have anti-cancer effects in pre-clinical and clinical studies. The anti-cancer effects of curcumin include inhibiting the carcinogenesis, inhibiting angiogenesis, and inhibiting tumour growth. This study aims to determine the Clinical effects of curcumin in different types of cancers using systematic review approach.

**Methods:**

A systematic review methodology is adopted for undertaking detailed analysis of the effects of curcumin in cancer therapy. The results presented in this paper is an outcome of extracting the findings of the studies selected from the articles published in international databases including SID, MagIran, IranMedex, IranDoc, Google Scholar, ScienceDirect, Scopus, PubMed and Web of Science (ISI). These databases were thoroughly searched, and the relevant publications were selected based on the plausible keywords, in accordance with the study aims, as follows: prevalence, curcumin, clinical features, cancer.

**Results:**

The results are derived based on several clinical studies on curcumin consumption with chemotherapy drugs, highlighting that curcumin increases the effectiveness of chemotherapy and radiotherapy which results in improving patient’s survival time, and increasing the expression of anti-metastatic proteins along with reducing their side effects.

**Conclusion:**

The comprehensive systematic review presented in this paper confirms that curcumin reduces the side effects of chemotherapy or radiotherapy, resulting in improving patients’ quality of life. A number of studies reported that, curcumin has increased patient survival time and decreased tumor markers’ level.

## Background

Research over the past 25 years has significantly increased our understanding of the molecular genetic basis of cancer. It is now well known that cancer is caused by a set of molecular genetic changes that lead to loss of growth control and cellular differentiation, resulting in uncontrollable cell growth that eventually leads to tumor formation [1].

More than half of all cancers occur in developing countries including those located in Southern America and Asia. Nearly three-quarters of people of these countries are classified into low or middle-income categories. The cancer survival rates in developing countries are generally one-third of the patients in the developed countries [2]. There are 9 million new cases of cancer each year, with 4 million new cancer cases in the developed countries and 5 million in developing countries [3]. In the next decades, cancer will be one of the leading causes of illness worldwide, and the number of new cases of various types of cancer is expected to rise to 15 million by 2020. Furthermore, cancer is predicted to be the leading cause of death by 2030 [4]. Given that cancer statistics are on the rise, and their treatments are costly, it is very crucial to find effective and economically viable methods for patients in low and middle income countries. Therefore, this study is motivated by using effective and relatively cheap treatments for cancer therapy. A systematic review of the clinical studies on curcumin use and its effectiveness in inhabiting and treating various types of cancer is carried out to obtain comprehensive information about the curcumin effects on cancer. Therefore, a structured review of all published articles and other relevant documents on the use of curcumin for cancer therapy creates a more complete picture of curcumin effects on cancer patients from different angles. In the process of this review only evidence from the studies with highest quality are selected to gather information and derive conclusion on curcumin effects and effectiveness at various stages of cancer therapy.

Among the medical herbs, Flavonoids are a large subgroup of the family of natural polyphenolic compounds that are the result of secondary metabolism in plants [5]. In recent years, research has shown that flavonoids have been very effective in the prevention and control of common diseases complex, such as cancer [6], cardiovascular diseases [7], Alzheimer’s [8], stroke, diabetes, Osteoporosis and rheumatoid arthritis. Furthermore, there are robust evidence of antiviral [9], anti-inflammatory [10] and anti-allergic effects [11] of flavonoids.

**Fig. 1:**
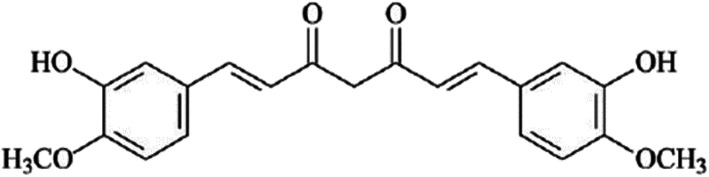
The Chemical expansion of curcumin [13], (By CorelDRAW Graphics Suite 22.1.0.517)

In the recent years, the use and effectiveness of medicinal herbs in treatment of various diseases has been received enormous attention. Huge research efforts were made on extraction and examination of the properties of the herbal compounds in the treatment of different types of diseases (e.g., cancers) and providing detailed mechanisms of drug performance of these compounds [12]. Amongst the wide range of the medical herbs, curcumin is an effective ingredient of turmeric plant with the scientific name of “longa Curcuma”, chemical name of “diferuloylmethane” and the chemical formula of C21 H20 O6 (as illustrated in Fig. 1) [14].

Curcumin makes up between 2 to 8% of turmeric compounds, and is considered as the main cause of yellow/golden colour of turmeric, and it has also been identified as responsible for many of the properties of turmeric [15, 16]. However, curcumin has low inherent toxicity and various properties with great impact and applications on a wide range of pharmacological developments, including antioxidant, anti-inflammatory, antimicrobial, and anti-cancer drugs [17–19].

Curcumin has been shown to have preventive and therapeutic effects on various types of cancers. The findings from several studies suggest that Curcumin compound can prevent the formation and spread of tumors or reduce their size. It was shown that curcumin can inhibit the formation of cancer and spread the cancerous cells by exerting anti-angiogenic effects, inducing apoptosis and interfering with the cell proliferation cycle [20, 21]. Curcumin exerts its anti-cancer effects through a variety of mechanisms. Curcumin inhibits and suppresses the proliferation of a wide range of cancer cells, which exerts its effects by reducing the modulation of anti-apoptotic gene products, activating caspase, and upregulating cancer-suppressive genes such as *P53* [22–24]. Recent studies confirm the preventive and therapeutic effects of curcumin on various types of cancers, indicating that it can prevent or reduce the formation or spread of tumors. Curcumin inhibits tumor invasion by reducing the modification of matrix metalloproteases (MMPs), the cell surface adhesive molecules *NF-κβ*, *AP*-1, *TNF-α*, *LOX* and *COX*-2, chemokines, growth factors (*HER*-2 and *EGFR*), inhibits *N*-Terminal activity and tyrosine kinase protein [21, 25, 26]. Curcumin inhibits angiogenesis in some tumors by suppressing angiogenic cytokines such as *IL*-6, *IL*-23, and *IL*-1*β* [27–29]. Due to the strong relationship between inflammation and cancer, the anti-inflammatory effects of curcumin would well result in its anti-tumor effects. It was reported that curcumin has prevented the development of several types of cancer by reducing the production of mediators of the inflammatory process, such as *COX*-2, lipoxygenase 2, *iNOS*, and related cytokines [27]. One of the possible mechanisms for suppressing tumor proliferation is the chemical inhibitor effect of curcumin. As a result, topical use of curcumin considerably inhibits inflammation due to tetradecanoylphorbol-13-acetate 12-*O*- (*TPA*), hyperplasia, cell proliferation, ODC activity, production of active oxygen species, oxidative DNA changes, and papillomavirus formation [30–32]. Multiple human gastrointestinal cell interactions with curcumin inhibit lipid peroxidation, inhibit *COX*-2 expression, inhibit *PGE*2 production, and increase glutathione-s-transferase enzyme levels [27, 33]. The other mechanism of the anti-cancer effects of curcumin, is due to its interference in the cell cycle, and reduction in CDK expression. CDKs are actually serine / threonine kinases that control cell cycle progression [34]. Furthermore, curcumin inhibits the *STAT*3 phosphorylation, which is responsible for signalling carcinogenic pathways [25].

Given that cancer statistics are on the rise, and their treatments are quite costly, it is very crucial to find some effective methods and economically viable for low and middle-income patients. Therefore, this paper provides up-to date evidence and findings of clinical studies on the effects of curcumin contributions in tumor cells survival and metastasis using a systematic review approach.

## Methods

Systematic review approach is adopted for undertaking this study by extracting the findings of the relevant studies selected from the articles published in national and international databases including SID, MagIran, IranMedex, IranDoc, Google Scholar, ScienceDirect, Scopus, PubMed and Web of Science (ISI). These databases were thoroughly searched, and the relevant publication records were selected based on the plausible keywords in accordance with the aim of this study, as follows: prevalence, curcumin, clinical features, cancer.

**Fig. 2:**
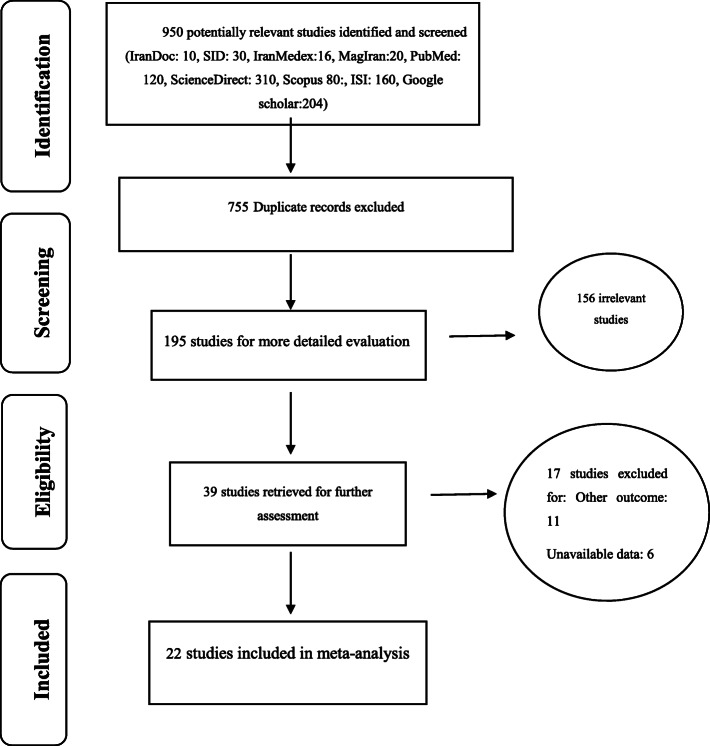
the flowchart on the stages of including the studies in the systematic review (PRISMA 2009)

The selection of relevant studies for the systematic review and the output quality control process involved several steps. First, all related articles were collected based on the search keywords mentioned. In the next step, the article specifications including the name of the journal and authors were hidden, and the full text of the articles were made available to the reviewers. Each article was investigated independently by two reviewers (MM, SHR) and if an article was excluded in the study, detailed rationale were give accordingly. In the case of disagreement between the two reviewers, the article was judged by a third reviewer. In this paper, all studies related to clinical investigations of curcumin use and impacts at various stages of cancer treatment, were systematically examined without any time constraints and according to PRISMA guidelines (Fig. 2).

### Article selection criteria

Articles with the following characteristics were selected for meta-analysis: original research articles, clinical trial studies, articles that their full text and data are available, and studies that examined the clinical effects of curcumin in various types of cancers. we prepared a list of articles specifications based on PRISMA 2009, including the researcher’s name, the article title, the year and place of the study, sample size and number of patients, duration of study, dosage of the drug and the result of the intervention (Table 1).

**Table 1:** examines the characteristics extracted in the studies

Author’s name, year	Country	Duration of study	Dosage of the drug	Number of patients	Results
Hejazi,2013 [35]	Iran	2 years	3 g per day	40	Curcumin reduces the severity of urinary symptoms.
Belcaro,2014 [36]	Italy	–	500 mg with soy lecithin	80	Curcumin reduced side effects.
Bayet-robert,2010 [37]	France	16 months	500 to 600 mg	10	Curcumin lowers the concentration of the CEA marker tumor.
Ryam,2013 [38]	USA	2 years	6 g	30	Curcumin reduces some skin complications.
Kanai,2011 [39]	Japan	11 months	8 g	22	Curcumin increased patient survival.
Hemati,2011 [40]	Iran	6 months	6 g	40	Curcumin reduces some skin problems.
Garcea,2005 [41]	UK	–	450 to 1800 mg per day	12	Curcumin increased the effectiveness of the colon.
Sharma,2001 [42]	UK	4 months	0.18–0.036 g per day	15	Curcumin reduced glutathione s-transferase activity.
Sharma,2004 [43]	UK	4 months	3.6–0.45 g per day	15	Curcumin reduces prostaglandin E2 production.
Cruz-correa M,2006 [44]	USA	5 months	1.44 g per day	10	Reduces the number and size of polyps without any significant toxicity.
Yu He,2010 [45]	China	10–30 days	1.08 g per day	126	Curcumin has been shown to improve the overall health of patients with colorectal cancer.
Durgaprasad,2005 [46]	India	6 Weeks	1.5 g per day	20	Curcumin reduced lipid peroxidation and increased glutathione content in patients.
Dhillon,2008 [47]	USA	–	8 g per day	25	Well-tolerated, limited absorption, and showed activity in some patients
Ide,2010 [48]	Japan	6 months	0.1 g per day	85	Reduced the serum prostate-specific antigen content in combination with isoflavones
Golombick,2009 [49]	Australia	6 months	4 g per day	26	Decreased para protein load and urinary N telopeptide of type I collagen
Polasa,1992 [50]	India	30 days	1.5 g per day	16	Reduced the urinary excretion of mutagens in smokers
Hastak,1997 [51]	India	3 months	3.6 g per day	26	Reduced the number of micronuclei in mucosal cells and in circulating lymphocytes
Cheng,2001 [52]	Taiwan	3 months	8 g per day	25	Improved the precancerous lesions
Rai,2010 [53]	UK	7 days	1 g per day	75	Increased vitamins C and E levels, decrease dmalondi aldehyde and 8-hydroxy deoxy guanosine contents in the serum and saliva
Marcia Cruz Correa,2018 [54]	New York	12 month	1500 mg orally, twice a day	44	No difference in polyp size and number between placebo and curcumin
Richard Greil,2018 [55]	USA	8 weeks	Doses between 100 and 300 mg per minute	32	No variation in tumor size according to RECIST criteria
Lynne M Howells,2019 [56]	United Kingdom	291 days	2 g per day	24	Curcumin was a safe and tolerable adjunct to FOLFOX chemotherapy in patients with metastatic colorectal cancer

### Article exclusion criteria

Studies including review papers, systematic review, meta-analysis, cohort, case-control, cross-sectional, descriptive and those which didn’t present samples from cancer patients and those which conducted with secondary data were excluded from the review. Duplicate publication and multiple publications from the same population will be removed using citation management, software EndNote (version X7, for Windows, Thomson Reuters).

### Quality assessment

The quality of the selected articles was evaluated based on criteria outlined by the CONSORT checklist; The last CONSORT statement, published in 2001, included 22 items. The CONSORT statement has been shown to improve the scientific quality of RCT reporting [57, 58], Each article was blindly assessed by two independent evaluators (MM, SHR). The result of each item was assessed by yes (1 point) or no (0 point), and some items were assessed as not applicable due to the features of studies. Accordingly, the maximum quality score of 22 was considered, and papers with a score of less than 12 were considered to have low quality, and thus they were excluded from the study.

### Curcumin role in the prevention of cancers

Free radicals and toxic products resulted from oxidative stress play an important role in the early stages of cancer formation. Therefore, compounds that have antioxidant effects can be helpful in preventing cancer formation. Curcumin has the property of trapping free radicals and thus can play a crucial role in inhibiting the onset of cancer. Several cellular and preclinical studies have reported that curcumin inhibits DNA damage caused by oxidative factors, such as ionizing radiation by inhibiting free radicals and active oxygen species [59]. The *NF*-*kappaB* plays an important role in the formation of Nitric oxide synthase and oxidative stress, and as a result causes cancer [59]. Curcumin suppresses the onset of cancer by inhibiting NF-kappaB from formation [59, 60]. Curcumin was reported to be effective on liver enzymes Cytochrome *p450* which has an imminent role in the oxidation and detoxification of toxins, it also inhibits the *Phase I* enzymes that is involved in the production of toxic metabolites and carcinogens. Furthermore, curcumin activates the *Phase II* enzymes, which plays a crucial role in detoxification of toxic metabolites [60]. Curcumin prevent tumor formation and growth by inhibiting and activating these two enzymes (*Phase I* & *II*) [60].

### Effect of curcumin on metastasis, angiogenesis and inflammation in cancer cells

**Fig. 3:**
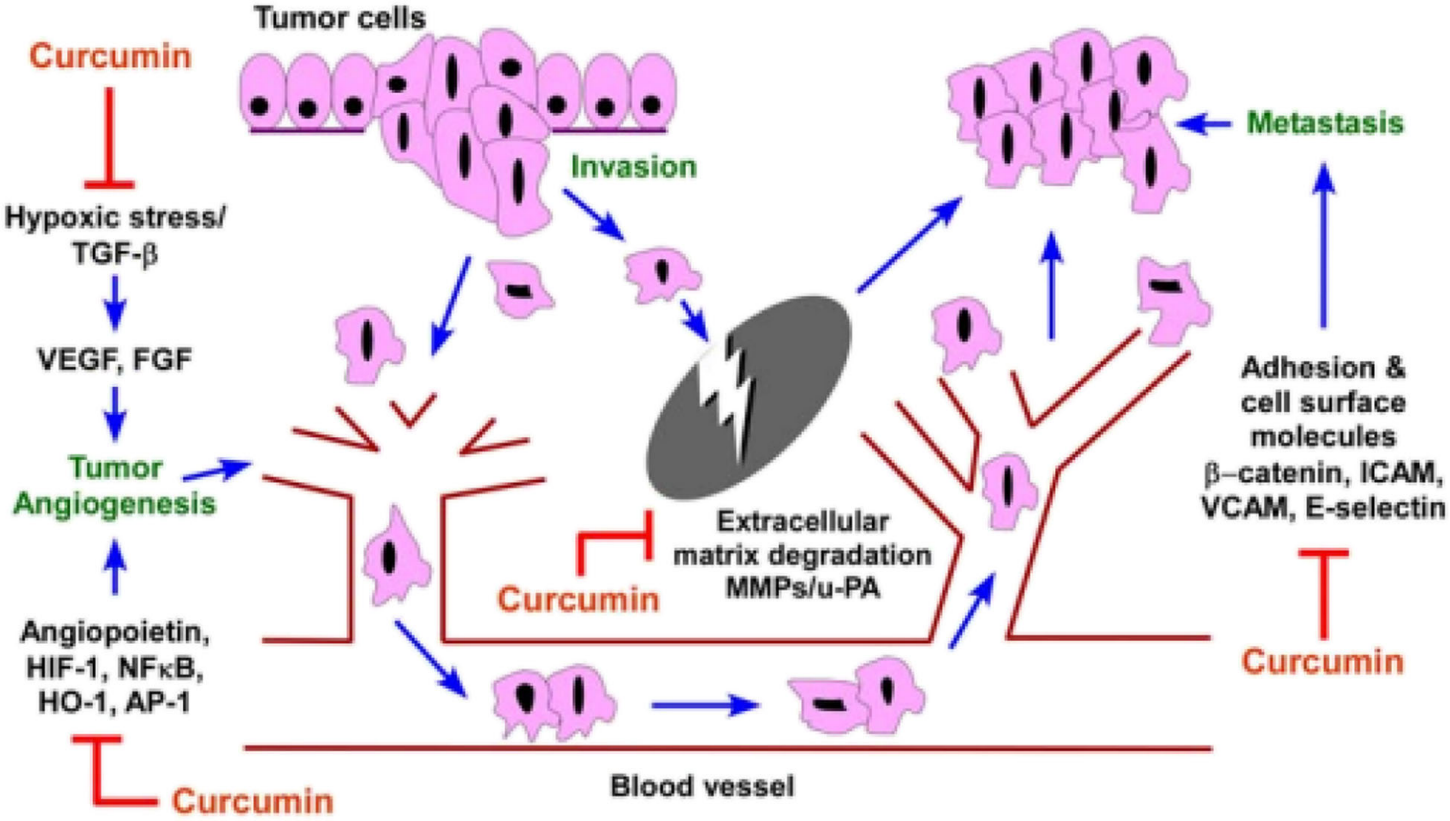
The effect of curcumin on angiogenesis and metastasis in cancer cells [60] (By CorelDRAW Graphics Suite 22.1.0.517)

Angiogenesis, is the process of new blood vessel formation from pre-existing vessels that is dependent on a price equilibrium between antiangiogenic and angiogenic factors. However, under pathological conditions, for example tumor growth, this tight regulation becomes lost which can result in tumor metastasis. Many gene products that are produced by different cells have a role in angiogenesis process. Hypoxia usually occurs in tumor sites. In order to overcome to hypoxia, tumor cells regulate and control the expression of genes related to angiogenesis, cell cycle, metastasis and drug resistance using hypoxia-inducible factor 1 (*HIF*-1). *HIF*-1 was first recognized as a transcription factor involved in hypoxia-induced erythropoietin expression. This factor has been presented as a main transcription regulator for these molecules [61, 62]. Several studies have shown that, *HIF*-1 activation of genes including vascular endothelial growth factor (VEGF), angiopoietin-1 (*Ang*-1) and angiopoietin-2 (*Ang*-2), *NF*-*KB*, etc., induced angiogenesis in the tumor cells. Furthermore, the activation of genes such as insulin-like growth factor 2 (IGF2), transforming growth factor a (TGF-a) and MAPK and *PI3K* signalling pathway will also enable the survival, proliferation and metastasis of tumor cells [63]. *HIF*-1 by activating genes involved in angiogenesis and also activates signalling pathways associated with cell survival and proliferation plays an important role in the stability and growth of tumors [64]. As above mentioned *HIF*1*α* is a potent activator of angiogenesis, and curcumin inhibits its expression. *AP*1 is a transcription factor that is activated in response to hypoxia, which is the principle physiological stimulus that induces angiogenesis. It is also involved in the conversion of epithelial cells to mesenchymal cells, which is the primary stage of metastasis, and causes the expression of MMP and *uPA* (Urokinase plasminogen activator) genes that are involved in tumor angiogenesis and its invasion. Curcumin inhibits the expression of this transcription factor [65]. Curcumin may inhibit angiogenesis directly by regulating angiogenic growth factors growth factors as well as the genes, including angiopoietin-1/− 2, *HIF*-1, *HO*-1, and transcription factors such as *NF*-*kappaB* (Fig. 3) [65–67]. It is known that hypoxic stress and activation of beta-growth factor (*TGF*-*β*) stimulate VEGF expression by activating *AP*-1 and the Hypoxia-inducible factors, *HIF*-(1) [68]. Curcumin is an important inhibitor in *AP*-1 activation, and it has recently been shown that curcumin is a direct inhibitor of HIF-1 transcription factor activity, which causes the transcription of many genes associated with angiogenesis in tumors [65, 69]. It is also shown that curcumin will reduce the expression of membrane surface molecules, including intracellular adhesion molecule-1, vascular cell adhesion molecule-1, and *E*-selectin, which play a role in cellular adhesion (Fig. 3) [68].

Curcumin affects a number of adhesive cellular molecules involved in tumor growth and metastasis processes [70]. Curcumin caused reduction in the expression of adhesive molecules inside the cells of (ICAM-1), VCAM (VCAM or Vascular cell adhesion molecule), and MMPs, which play an important role in cellular adhesion and metastasis [71]. Furthermore, curcumin results in increase of the expression of various anti-metastatic proteins, including tissue inhibitor metalloproteinase (TIMP 2), the non-metastatic gene NM23, and E-cadherin. Lack of E-cadherin would increase the possibility of metastasis. Because *E*-cadherin are essential to maintain cellular adhesion [72]. Angiogenesis is also linked with neoplasia. Angiogenesis means the formation of new blood vessels, which is generally a major step in tumor survival and growth. Curcumin inhibits cancer in various organs [61–64, 73].

The anti-inflammatory effects of curcumin have been proven in many studies. Since, oxidative stress leads to chronic inflammatory diseases, antioxidant compounds can be useful in the prevention and treatment of inflammatory disorders [60, 65, 70, 74]. On the other hand, since Curcumin has a high antioxidant activity, it will not be easy to assess whether curcumin’s anti-inflammatory activity is also dependent on its antioxidant activity [65, 74]. Since many of the antioxidants that have been already identified do not have anti-inflammatory properties, it seems unlikely that the anti-inflammatory effects of curcumin are due solely to its antioxidant properties. Curcumin, as a potent anti-inflammatory factor, expresses its own effects through several mechanisms. First, curcumin inhibits the activation of the NF-κβ factor [65, 70, 71, 74].

The Lab based studies have revealed that curcumin neutralizes oxidative stress caused by tumor and restores *NFkappaB* activity. Curcumin inhibits *TNF-α* production, thus *T*-cell apoptosis caused by tumor will be minimised [74].

## Results

In the initial screening of databases, 950 articles were identified, after deleting duplicate articles, 195 studies were obtained. After deleting 156 unrelated articles, 39 studies were obtained17 articles were also deleted due to lack of access to their full-text or falling into the low quality category. At the end, 22 studies entered the final phase and analysis as illustrated in Fig. 2. The specifications and details of the studies considered in this systematic review are summarized in Table 1.

According to the studies presented in Table 1, curcumin has reduced side effects, including skin complications. Depending on the different doses of curcumin prescribed for the patients suffering from cancer, their survival rate was increased and their symptoms of chemotherapy were reduced. In studies examining the effect of curcumin on colorectal cancer, curcumin has increased efficacy in the large intestine, reduced glutathione *s*-transferase activity, and reduced prostaglandin *E*2 production. Curcumin also reduces the number and size of polyps without any significant toxicity. Curcumin in pancreatic cancer reduces lipids’ peroxidation and increases glutathione content in the patients with this type of cancer. In prostate cancer, curcumin reduces the serum levels of prostate-specific antigen in combination with Isoflavones, and also reduces the severity of urinary symptoms. According to the published studies, the use of curcumin during radiation therapy for breast cancer patients improved treatment outcomes for these patients, such as preventing skin symptoms, reducing pain and suffering of patients, improving their quality of life during treatment, and reducing delays or unwanted stops during the course of radiation therapy. Curcumin can regulate multiple signalling pathways and affect different molecular targets. Low cost, pharmacological safety, efficiency, and multiple molecular targets make curcumin a promising product for the prevention and treatment of various human diseases (Table 1).

After collecting various articles from reputable databases, and deleting duplicate articles and removing unrelated articles to the main aim of this paper, we finally considered 22 articles for further investigation and analysis. The main aim of this paper is to review the clinical studies about curcumin and its various purposes/effects on cancer. The results reported from numerous clinical studies that have examined the effects of curcumin on the patients who are suffering from cancer, and undergoing radiotherapy and chemotherapy, were very promising. Here, we briefly describe some of these studies, which are summarised in Table 1.

Garcea et al. (2005) evaluated 12 patients in the UK. In this study, each patient received 450 to 1800 mg of curcumin per day. At the same time that these patients received curcumin, they were treated with radiotherapy and chemotherapy. The results of this study revealed that curcumin increased the effectiveness of the treatment plan for colorectal cancer in the patients received with curcumin [41].

In 2010, Bayet-Robert conducted a study, consists of fourteen patients with advanced breast cancer who were being treated with docetaxel chemotherapy and simultaneously received curcumin at different doses up to a maximum dose of 8 g per day for 7 days per each treatment cycle. Finally, 10 patients participated in this study were able to complete this treatment plan. Nutropenia and leukopenia were the most important toxicities caused by docetaxel administration after 8 days. Two patients refused to continue treatment because they received 16 curcumin capsules, however curcumin treatment continued by reducing the dosage to a maximum of 6 g per day. Nine patients were screened for tumor response. Six weeks after completing the course of treatment, 5 patients partially responded well, but three patients still suffered from the disease. In this study, the CA 15.3 tumor marker did not decrease, but the CEA tumor marker decreased compared to the initial value prior to the treatment. In 8 patients, the VEGF (Vascular endothelial growth factor) as a tomur marker, which indicates tumor growth, metastasis, and malignancy, was reduced by 30% compared to the baseline before treatment [37].

In 2014, the effect of curcumin on reducing the side effects of radiotherapy and chemotherapy in patients with ovarian, lung, colon, liver, kidney, and stomach cancers was investigated. Eighty patients received 500 mg of curcumin simultaneously with radiotherapy. The duration of this study was 60 days. The incidence of side effects such as nausea, diarrhea, constipation, and weight loss decreased in patients who treated with both radiotherapy and curcumin. In the patients who are simultaneously under radiotherapy and received curcumin, the prevalence of side effects such as skin lesions, mouth and throat ulcers, swallowing problems, nausea, vomiting, fatigue, weakness and common medications required for treating side effects were statistically lower than the control group [36].

In a study conducted by Hemati in Iran, 40 patients receiving radiation therapy to the breast area due to breast cancer, from 2 days before the start to the end of radiotherapy, 4500 mg-capsules containing curcumin were taken orally 3 times a day [40].

Yu He et al. evaluated 126 patients in their study and stated that a dose of 1.08 g of curcumin per day for 10–30 days improved the general health of patients with colorectal cancer through increasing the expression of P53 molecules in tumor cells [45]. In a study conducted by Cruz-correa, it was stated that a dose of 1.44 g of curcumin per day will reduce the number and size of polyps without any significant toxicity [44].

## Discussion

In the recent years, several studies have been conducted on the biological effects of curcumin. In more than 3000 studies, have been recently published, curcumin has shown to have various effects in cancer treatments. Curcumin has antioxidant, antibacterial, antifungal, antiviral, anti-inflammatory, anti-proliferative, pro-apoptotic effects, etc. Curcumin has tremendous potential for treatment of neurodegenerative diseases, arthritis, diabetes, psoriasis, allergies, intestinal inflammation, kidney poisoning, Alzheimer’s, depression, AIDS, multiple sclerosis, cardiovascular disease, and especially cancer [75–78]. The numerous and multifaceted effects of curcumin in determining the cellular targets and molecular mechanisms involved in curcumin pathways have attracted much attention from researchers. Curcumin is a multifaceted molecule and has many therapeutic effects. The multifaceted effects of curcumin are due to its capacity to interact with different molecules, and to regulate multiple molecular pathways and their targets [79].

One of the compelling properties of curcumin, which makes it appropriate for therapeutic use, is its low toxicity, so that even its consumption up to a dose of 10 g per day does not cause any side effects [80]. Consumption of curcumin in high-dose prevents cancer cells from multiplying, although it does not damage healthy cells [81, 82].

Minimal toxicity alongside with possessing many therapeutic effects have led to the widespread use of natural plant-derived compounds in the treatment of cancer [83]. The compounds found in nature target various cellular and molecular aspects of cancer cells [84]. The researchers have demonstrated that curcumin regulates signalling pathways in cancer cells, reduces the expression of proteins related to drug resistance and increases the performance of anti-tumor drugs at various levels. Curcumin reverses drug resistance mechanisms and results in increasing the sensitivity of chemotherapy-resistant cells. In the research conducted by Keyvani-Ghamsari et al., they demonstrated that curcumin is an effective chemical in cancer treatment [85].

In laboratory studies, which have been performed on the cellular categories of colorectal cancer, the derived results show that curcumin inhibits cell growth, and also stimulates apoptosis by interacting with several molecular targets. Furthermore, curcumin has been used as part of dietary formulations to prevent colon cancer. In vitro and in vivo, these compounds have been shown to have anti-cancer properties for colon cancer and its inflammation. The results of this study show that curcumin would be effective in preventing colorectal cancer in animals. This property offers promising expectations in humans. Due to the limited number of the human clinical studies, the corresponding results are somehow contradictory. On the other hand, there exist several unanswered questions about dosage, bioavailability, optimal signs, and potential toxicity which should be investigated in future studies using sufficiently large samples [86]. In addition, curcumin can induce autophagy, apoptosis, and cell cycle arrest, in order to reduce the survival and proliferation of lung cancer cells. Curcumin has this promising capability to increase the effectiveness of radiotherapy in the treatment of lung cancer by targeting different signalling pathways such as epidermal growth factor receptor and NF κB. Curcumin-containing nanocarriers increase bioavailability, cell uptake, and curcumin antitumor activity [87, 88].

In a study conducted by Cruz-Correa et al., oral curcumin was prescribed to the patients with adenomatous polyposis. This research was implemented to determine the safety and efficacy of curcumin in patients with adenomatous polyposis. In this study, 1500 mg of oral curcumin was administered twice per day over 12 months, to 44 patients with adenomatous polyposis. The results showed that there was no significant difference between those who received oral curcumin and those receiving placebo [54]. In another study conducted by Grell et al., 32 patients were subjected to receive doses between 100 and 300 mg per minute. The main aim of their study was to evaluate the safety of curcumin locally in patients with advanced or metastatic cancer. The results obtained from their study showed that no change in tumor size was observed based on the Recist criteria [55]. In 2019, Howells et al. evaluated 24 patients with the age over 18 and with metastatic colorectal cancer using the histological diagnosis. Quality of life and neurotoxicity of these patients were assessed using questionnaires. The derived results showed that curcumin is a safe and tolerable adjunct for FOLFOX chemotherapy in patients with metastatic colorectal cancer [56].

Overall, the results suggest that curcumin can be used as an effective combination in inhibiting and controlling cancers, improving clinical symptoms and preventing tumor spread and metastasis. This compound would affect various molecular pathways and inhibits vasodilation, cell proliferation, and metastasis.

## Conclusion

Curcumin is a natural product found in turmeric that has a unique chemical structure, with particular biological and medicinal properties. Through various cellular and molecular mechanisms, curcumin inhibits the carcinogenesis and their growth. Due to the fact that no specific toxic effects of this natural product have been reported, its use has been considered as a drug supplement in therapeutic diets of cancer patients. In a number of studies considered in this systematic review have shown that taking curcumin would increase the expression of anti-metastatic proteins. In several other studies, it was reported that curcumin has also increased patient survival and decreased tumor marker concentration.

## Data Availability

Datasets are available through the corresponding author upon reasonable request.

## Abbreviations

MMPs: matrix metalloproteases
VCAM: Vascular cell adhesion molecule
WHO: World Health Organization
SID: Scientific Information Database
PRISMA: Preferred Reporting Items for Systematic Reviews.
